# Importance of hypersensitivity in adverse reactions to drugs in the elderly

**DOI:** 10.1186/s12948-018-0083-x

**Published:** 2018-04-02

**Authors:** Maria Teresa Ventura, Elisa Boni, Rosa Cecere, Rosalba Buquicchio, Gian Franco Calogiuri, Irene Martignago, Cristoforo Incorvaia, Erminia Ridolo

**Affiliations:** 1 Department of Interdisciplinary Medicine, University of Bari, Bari, Italy; 2 S.O.S.Allergy, ASL, Alessandria, Italy; 3 Pneumology Department, Ospedale Sacro Cuore Gallipoli (Lecce), Lecce, Italy; 4 Medicine and Surgery Department, University of Parma, Via Gramsci 14, 43126 Parma, Italy; 5 Cardiac/Pulmonary Rehabilitation, ASST Pini/CTO, Milan, Italy

**Keywords:** Drug allergy, Elderly, Adverse drug reaction, Hypersensitivity drug reaction

## Abstract

**Background:**

The use of drugs in the elderly is very extensive because of the frequent occurrence of chronic diseases. Adverse drug reactions (ADRs) commonly occur in geriatric patients receiving multiple therapeutic regimens. In the literature, little attention has been given to ADRs in the elderly, and particularly to allergic reactions.

**Objective:**

The aim of the present study is to provide data on possible inappropriate prescriptions in the elderly in relation to allergic reactions and to identify a list of drugs which are likely inducers of allergic reactions.

**Methods:**

We retrospectively evaluated ADRs in patients referring to Immunoallergy Unit of Hospital Policlinico in Bari on the basis of Beers criteria. Among adverse reactions, hypersensitivity reactions were extracted and a comparison between different age groups was assessed.

**Results:**

Out of 823 patients with ADRs, in 30.6% hypersensitivity drug reactions (HDR) were diagnosed. Data about drug intake, comorbidities and clinical presentation were collected, aiming to identify possible risk factors. An evaluation of drugs most commonly involved was assessed.

**Conclusions:**

HDR are reported to represent 5–10% of all ADRs, while in our study population the prevalence was about 30%. This suggests the need to develop strategies to minimize the incidence of drug allergy in the elderly, as well to reduce the phenomenon of inappropriate prescriptions.

## Background

The concomitant use of several drugs in the elderly is closely related to the multiple chronic diseases typical of this age. Moreover, polypharmacy determines an increased risk of adverse reactions that can be related to drug–drug or drug-disease interactions [1]. In fact, the percentage of adverse events increases from 13%, in case of concomitant intake of two drugs to 82% in case of intake of 7 or more drugs [2]. Another relevant factor is the alteration in the pharmacokinetics (absorption, distribution, metabolism, excretion) and pharmacodynamics, as well as the changes in the body mass in terms of total body fat and water [3]. Of consequence, the hydrophilic drugs may be overdosed, while lipophilic drugs cannot perform to their full action. Modifications in drug metabolization and excretion, related to liver failure, as well as renal insufficiency should not be neglected [4]. Finally, the decline of the oxidation of cytochrome P450 causes additional problems not only for the consequences of this partial enzyme depletion in terms of drug–drug interactions [5], but also in terms of accumulation of reactive metabolites [6].

From a clinical perspective, adverse drug reactions (ADRs) are often difficult to diagnose and are sometimes mistaken as a new disease and treated with additional medications. Discontinuation of previously prescribed therapy in the elderly is rarely considered. As a result, the older population receives a considerable quantity of doses per day of different pharmacological principles. According to Edwards et al., ADRs are classified as follows: type A (dose-dependent), which are quite frequent, related to the mechanism of action of the molecule, predictable and usually associated with low mortality; type B (bizarre, not dose-dependent), rare and unpredictable with a high mortality, such as hypersensitivity reactions; type C (chronic, dose- and time-dependent), related to the repeated treatment; type D (delayed), rare and generally dose-dependent, occur sometimes following the use (e.g. carcinogenesis); type E (end of use) occur immediately after the suspension; type F (failure), characterized by therapeutic ineffectiveness, dose-dependent and often caused by interactions between drugs [7].

The European Academy of Allergy and Clinical Immunology (EAACI) Nomenclature Task Force introduced a classification of ADRs. The reactions of hypersensitivity to drugs have been divided into two subgroups depending on the genesis of immunological (drug allergy) or non-immunological hypersensitivity (non allergic). Drug allergy is in turn divided into IgE-mediated and non-IgE-mediated [8]. The reactions previously defined pseudoallergic reactions (PAR) and now instead referred to as non allergic [9], that consist of clinical manifestations similar to those induced by allergic reactions, are due to one or more of the following pathogenetic mechanisms: (1) inhibition of cyclooxygenase with reduced production of prostaglandins and production of inflammatory leukotrienes. Responsible drugs are: acetylsalicylic acid (ASA), nonsteroidal anti-inflammatory drugs (NSAIDs), analgesics, barbiturates, antihistamines, sulfonamides, quinine, some local anesthetics, hydrocortisone; (2) direct activation of complement with release of anaphylatoxins able to induce mast cell degranulation. This mechanism is frequent after the administration of iodinated contrast agents, adrenocorticotropic hormone, endovenous anaesthetics, endovenous globulins, plasma substitutes; (3) direct release of histamine due to mast cell instability in predisposed people. Drugs that can provoke this mechanism are morphine, codeine, aminoglycosides, plasma substitutes, antidepressants, sympathomimetics, pesticides; (4) bronchospasm due to the liberation of sulphur dioxide in the course of sulphide therapy or drugs containing sulphites; (5) inhibition of degradation and subsequent accumulation as it occurs following prolonged Angiotensin Converting Enzyme (ACE)-inhibitors assumption. The issue of polypharmacy in the elderly has been reviewed by several authors. Beers et al. [10] in 1991 published an article that contains the “criteria” to be adopted for the management of the therapy in the elderly, in addition to a list of drugs to be avoided at this age. These criteria were then revised by several authors [11–14]. The Beers criteria comprise practical guidelines widely adopted and able to identify inappropriate prescriptions in the elderly. The 2003 update expanded the list of medication and reviewed the comorbidities including cognitive impairment, incontinence and the depressive syndrome associated with Parkinson’s disease [14]. Despite the comprehensiveness of Beers’s work [10, 12] and the subsequent updates with modifications and implementation of the list of drugs, it should be noted that, while the ADRs of type A and C and interactions are exhaustively included, the hypersensitivity reactions—allergic or non allergic—according to the classification proposed by the EAACI Nomenclature Task Force in 2001, are missing [8, 13].

## Methods

The aim of the present study was to evaluate the possibly inappropriate prescriptions in the elderly in relation to allergic reactions and to identify a list of drugs which are potential inducers of allergic reactions to be avoided in the elderly. This is to prevent the risk of IgE- or cell-mediated reactions or potential cross reactions between medications with similar immunological mechanisms. In the study were retrospectively included patients who suffered of drug reactions visited between 2011 and 2012 in the Immunoallergology Unit in Hospital Policlinico in Bari. ADRs were diagnosed on the basis of Beers criteria [10–14]. Diagnosis of hypersensitivity drug reaction (HDR) was made based on the clinical history collected through a questionnaire assessing clinical manifestations, chronology of the symptoms, other medications taken, and the medical background of the patient [15]. The diagnostic process was completed, when indicated, with in vivo and in vitro allergy testing according to EAACI criteria (Table 1) [16]. In case of suspected IgE-dependent reactions to penicillins, cephalosporins, insulin, heparin, contrast media and general and local anesthetics, in vivo tests (skin prick test and/or intradermal test) for the culprit drug were performed if available. Moreover, depending on the drug involved, in vitro tests were performed, including Basophil Activation Test (BAT) (Buhlmann Lab., Basel, Switzerland), specific IgE (CAP system, Thermo Fisher Diagnostic, Uppsala, Sweden), determination of complement factors and circulating immune complexes, measurement of histamine and tryptase in serum (CAP FEIA Thermo Fisher Diagnostic). Drug provocation test with the culprit drug is not a routine test in our Unit, due to age and comorbidities of patients; for the same reason, in case of hypersensitivity reactions to contrast media, provocation test was not performed. An alternative contrast media was indicated with premedication in case of further imaging investigations. An oral challenge test with the various medications was instead performed to find an alternative treatment. In the case of topical medications, the diagnosis was assessed by mean of patch tests [16].

**Table 1:** Tests for the diagnosis of hypersensitivity to drugs

Type of drug	In vivo tests			In vitro tests	
	Prick test/intradermal test	Patch test	Provocation Test	Specific IgE	Others
Penicillins	+	+	+*	+	
Cephalosporins	+	+	+*	+	
Sulfonamides			+		
Other antibiotics			+		
Aspirin and NSAIDs			+		+
General anesthetics	+		+*		
Local anesthetics	+		+*		
Contrast media	+	+			
Insulin	+			+	
Heparin	+	+	+*		
Immunoglobulins					+
Anticonvulsants			+		

*performed after cutaneous test

With regard to patients with history of HDR, data about gender, smoking habit and use of self-medications was collected. Other clinical data of interest concerned family medical history, atopy history, presence of concomitant diseases (liver diseases, renal failure, cancer, cardiovascular diseases, diabetes, chronic respiratory diseases). Special attention was paid to dose assumption, clinical presentation and severity of HDR, previous reactions to drugs, and current pharmacological regimen. The patients with HDRs were then divided into two subgroups (under 65 and over 65 years) and the data collected were analyzed for each age group.

## Statistical analysis

Age, which is a continuous quantitative variable distributed according to Gauss, has been synthesized as mean and standard deviation. Later it was categorized into two classes: above and over 65. This and the other variables object of the analysis that are all qualitative, are summarized as counts and relative percentage. The comparison of the percentages between independent groups was carried out by means of the Chi square test.

## Results

**Fig. 1:**
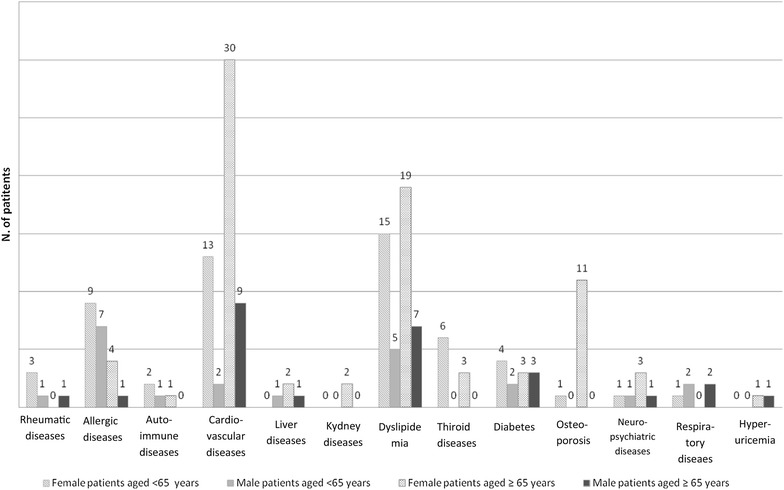
Comorbidities in patients with hypersensitivity drug reactions

**Fig. 2:**
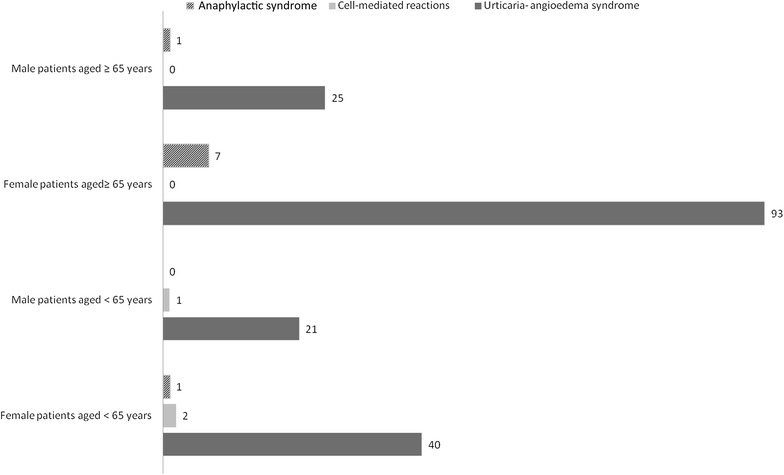
Clinical manifestation of hypersensitivity drug reactions in different age groups and gender

From 2011 to 2012, 1782 patients were visited in our Unit. Of them, 823 (mean age 45 ± 4 years, 543 females and 280 males) were included in the study for suspected ADRs: the diagnosis of ADRs was confirmed in 623 cases (mean age 66 ± 3). Among patients with ADRs, 421 were females (67.6%) and 202 were males (32.4%). The median time of onset of symptoms was about 10 h after drug intake and in most patients the symptoms were related to pharmacological mechanisms of the drug (e.g. syncope after antihypertensive therapy, diarrhea after use of antibiotics, hypotension due to high dose of diuretic, etc.). In 191 patients (30.6%), 143 females and 48 males, HDR was diagnosed on the basis of clinical manifestations and specific allergy tests; 126 patients (66%) were aged ≥ 65 years and 65 (34%) were aged < 65 years. Comorbidities are shown in Fig. 1, they reflected the common distribution in the general population of age-associated diseases. Demographic and medical data of patients who suffered from HDR are reported in Table 2: only 3.9% of geriatric patients had other allergic diseases, compared with 24.6% among younger patients, confirming that there is no correlation between atopy and HDRs in older patients. There were no significant differences between familial history of atopy, smoking habit and alcohol abuse in the two age groups. The prevalence of HDRs was significantly higher in older female patients compared to younger patients (80.15% vs. 64.61%), as well as in the older group concerning multiple chronic therapeutic regimen (86.5% vs 30.7%) and in short course therapies with 3 or more drugs (38% vs 27.6%). With regard to the different clinical presentations of HDRs, as shown in Fig. 2, skin symptoms were the main clinical manifestation of HDRs in both age groups, with urticaria-angioedema syndrome as unique presentation in more than 90% of patients of every age. All immediate reactions appeared within 1–5 h after intake of a drug. On the other hand, non immediate T cell reactions occurred only in 3 patients under the age of 65. Rare manifestations were erythema multiforme, Stevens Johnson syndrome and psoriasis-like eruptions (data not shown). Anaphylactic shock occurred in 7% of patients aged over 65 vs. 1.5% of younger patients. Finally, in Table 3 are listed the drugs responsible for HDRs. Over the age of 65 years ASA and other NSAIDs were the main cause of drug reactions, followed by antibiotics. Conversely, antibiotics were the first cause of HDR in younger patients and none of them had reactions to ASA or NSAIDs.

**Table 2:** Demographic and medical data of patients who suffered from HDR

Feature	Patients aged ≥ 65 year n. (%)	Patients aged < 65 year n. (%)	Χ^2^
Female	101 (80.15%)	42 (64.61%)	0.019
Family history of atopy	0	4 (6.1%)	0.005
Allergic diseases	5 (3.96%)	16 (24.61%)	0.000
Previous ADRs	12 (9.5%)	15 (23.07%)	0.01
Smoker	38 (30.15%)	22 (33.8%)	0.603
Alcohol abuse	19 (15%)	6 (9.23%)	0.256
Multiple chronic drug regimen	109 (86.5%)	20 (30.7%)	0.000
Comorbidities	126 (100%)	6 (9.23%)	0.000
Use of self-medications	31 (24.6%)	22 (33.8%)	0.176
Short course therapy with ≥ 3 drugs	48 (38%)	18 (27.6%)	0.152
Total	126 (100%)	65 (100%)

**Table 3:** Drugs responsible for HDRs

	Patients aged ≥ 65 year		Patients aged < 65 year	
	Females % of pts ≥ 65 (n.)	Males % of pts ≥ 65 (n.)	Females % of pts < 65 (n.)	Males % of pts < 65 (n.)
Antibiotics	33.3 (42)	7.9 (10)	46.1 (30)	23 (15)
NSAIDs	35.7 (45)	7.9 (10)	7.6 (5)	1.5 (1)
Acetylsalicilic acid	11.9 (15)	7.1 (9)	0 (0)	0 (0)
Antihypertensive drugs	1.5 (2)	0 (0)	6.1 (4)	6.1 (4)
Antiacids	4.7 (6)	0 (0)	1.5 (1)	0 (0)
Psicotropic agents	3.1 (4)	0.7 (1)	13.8 (9)	0 (0)
Anesthetics	3.1 (4)	0 (0)	0 (0)	1.5 (1)
Corticosteroids	3.9 (5)	0 (0)	0 (0)	0 (0)
Allopurinol	1.5 (2)	0.7 (1)	0 (0)	0 (0)
Antimycotic	0.7 (1)	1.5 (2)	0 (0)	0 (0)
Contrast media	3.1 (4)	0.7 (1)	0 (0)	0 (0)
Heparin	2.3 (3)	0.7 (1)	0 (0)	1.5 (1)
Statins	1.5 (2)	0.7 (1)	0 (0)	0 (0)
Bone metabolism drugs	1.5 (2)	0 (0)	0 (0)	0 (0)
Drugs for gastrointestinal diseases	1.5 (2)	0 (0)	1.5 (1)	0 (0)
Anticoagulants	0.7 (1)	0 (0)	1.5 (1)	0 (0)
Muscle relaxant	0.7 (1)	0 (0)	1.5 (1)	0 (0)
Plasma expander	0.7 (1)	0 (0)	0 (0)	0 (0)
Iron therapy	0.7 (1)	0 (0)	0 (0)	0 (0)

## Discussion

HDRs, previously called type B or bizarre [17] are not directly related to the drug action but are individual responses in predisposed patients. These reactions are defined as quantitatively and qualitatively abnormal, unpredictable and often dose-independent. Concerning their pathogenesis, they are distinguished between non-immune and immune (or allergic). HDRs are less frequently described in the literature with respect to the ADRs in the elderly. It is well established that only certain drugs or biological agents have the structural characteristics of immunogenicity, being able to trigger immunopathological responses. Most drugs or their metabolites, however, have no such immunogenic properties. Therefore, in order to elicit an immune response, a phenomenon of haptenization is necessary. A typical example is provided by β-lactam antibiotics that have high reactivity with carrier proteins, able to induce an IgE-mediated reaction in predisposed patients. Moreover, there are drugs potentially able to induce clinical manifestations similar to allergic reactions, though immunological mechanisms are other than IgE-mediated reaction: ASA and NSAIDs, ACE inhibitors, opioids, iodinated contrast media, antibiotics such as ciprofloxacin and vancomycin, and muscle relaxants with ammonium group. It is known that allopurinol is able to elicit an hypersensitivity syndrome characterized by severe skin involvement, worsening of renal function with fever, leukocytosis and eosinophilia that can be fatal [18]. It recognizes an immunological genesis determined by T lymphocyte clones specific for its metabolite, oxypurinol. Risk factors for the development of this syndrome are represented by an age ≥ 65 years, chronic renal insufficiency, concomitant treatment with thiazide diuretics and intermittent therapy with allopurinol [19, 20]. HDRs represent 5–10% of all adverse reactions to drugs [21], but as they are unpredictable and potentially lethal, they represent the most important kind of adverse reactions both in youth and elderly. In our study population the rate of HDRs was 30%, that is much higher than previously reported, presumably due to the fact that in our area many patients affected by ADRs are not referred to specialist’s evaluation. Risk factors related to patients are female gender, previous reactions to medications, genetic factors such as familiarity for drug allergy, association with particular MHC antigens, acetylator phenotype, and a personal history of atopy [22]. Other risk factors comprise concomitant diseases such as viral infections, chronic liver disease or chronic renal failure [23]. Moreover, renal failure not clinically apparent may result in an increased risk of ADRs, especially in water-soluble drugs, such as insulin and glibenclamide [4, 24]. According to Beyth, the risk is correlated with the number of drugs taken and with the number of pathologies, as well as with the nutritional status and with alterations of pharmacodynamics and pharmacokinetics of the molecule involved [25]. In the present study we observed that gender as well as multiple therapeutic regimens are risk factors in older age for HDRs, with an higher rate than patients aged under 65 years. Smoking habit, alcohol abuse and personal and familiar history of atopy did not differ between younger and older subjects. Atopy and previous reactions to drugs do not seem to represent risk factors in the elderly, while we observed a positive association with multiple drugs intake. This offers confirmation to previous observations about the lack of association of atopy and HDRs [26], although this could be influenced by the severity of reactions in atopic subjects [27]. The main clinical manifestation according to our data is skin involvement, as previously described in a study conducted on a cohort of 2644 subjects. In that study, after allergy testing HDRs were diagnosed in 2.1% of cases; 96% of symptoms were cutaneous (urticaria, angioedema, erythema, Steven Johnson syndrome, and psoriasis-like reactions). It was also observed that the peak of HDRs dramatically decreases in the eighth decade of life, with significant prevalence in females (80%). In the same study, comparing the two groups of patients (elderly and aged < 65 years), the family history and personal history of atopy was similar, comorbidities and multiple treatment regimen were instead a peculiarity of the elderly [26]. Some authors have found that HDRs are less frequent and less severe in children and in elderly, probably due to immaturity and involution of the immune system typical of these periods of life [28]. This is in contrast with our data that shows that anaphylactic shock is more frequent in > 65 years aged than in younger patients. In addition, in the present study antibiotics, medications for cardiovascular disease, NSAIDs and ASA were the drugs most frequently responsible for HDRs in the elderly. We observed that ASA and NSAIDs are the main factors of drug reactions in > 65 years aged while antibiotics are the main factor in < 65 years aged patients. Some chronic degenerative diseases such as atherosclerosis are favored by inflammatory phenomena that tend to induce chronic vascular endothelial lesions typical of this disease. A common mechanism appears to contribute to atherosclerosis and atopy [29]. The activation of perivascular mast cells promotes atherosclerosis by the release of inflammatory cytokines that contribute to the activation and chemotaxis of macrophages, which incorporate the excess fats becoming “foam cells” (fatty cells) and generate the atherosclerotic plaque [30]. Similarly, it is believed that leukotrienes play an important role in promoting atherosclerosis. A genetic variant of the 5-lipoxygenase has been associated both with asthma and atherosclerosis [31]. In turn, the inflammatory cytokines released by mast-cells and macrophages in the genesis of the atherosclerotic plaque can form the protein component to which the hapten drug can bind, even in absence of albumin, the production of which by the liver is always reduced in the elderly [32]. A recent study has also highlighted that the increased DNA methylation predisposes the elderly to respiratory allergic diseases, but could also predispose to HDRs [33]. The misuse of complementary and alternative medicine and polypharmacy during different degenerative diseases afflicting the elderly is another risk factor for the development of drug allergy [34]. A recent retrospective survey on 161 hospitalized patients (mean age 63 years) who had an HDR to medications during their hospital stay suggested that the administration of proton pump inhibitors (PPI), used in 83% of hospitalized patients, could favor the occurrence of HDRs, especially to NSAIDs. In fact, with the suppression of gastric acid, PPIs increase the persistence of labile proteins able to bind stably to other drugs taken by oral route, thus preventing the complete denaturing of the other drugs [35]. Similarly, in a case report a patient aged 60 years developed generalized urticaria immediately after a second course of therapy with diclofenac and PPI, the latter having been used previously for gastroesophageal reflux. The authors postulated that allergy to diclofenac could have been favored by suppression of gastric acid and confirmed it on a murine model of sensitization [36]. Considering the extensive use of PPIs as gastroprotective drugs in older patients treated with low dose ASA as a preventive therapy of myocardial ischemia, one could speculate that PPI might represent a co-factor in HDRs to ASA. In conclusion, the purpose of this study was to find data able to suggest the basis to develop strategies to minimize the incidence of HDRs in the elderly, as the Beers criteria have served to reduce significantly the phenomenon of inappropriate prescriptions in this time of life. According to the results, the comorbidity and proinflammatory status of the immune system, typical of the elderly, such as metabolic disorders, cardiovascular diseases, and osteoporosis may favor the onset of HDRs. In these patients not only beta-lactam antibiotics, able to elicit IgE-mediated responses, but also drugs that are able to elicit non IgE or cell-mediated drug reaction, as ASA, NSAIDs, ACE inhibitors or cyclooxygenase and bradykinin inhibitors have to be administered with caution in older patients. At the same time, in order to re-evaluate the issue of HDRs in the elderly and to establish shared parameters about the administration of drugs at risk of reactions, responsible for anaphylaxis or urticaria-angioedema syndrome, the incidence of HDRs in geriatric age and the risk factors for this type of reactions should be estimated. Based on adequate data, guidelines for the management of HDRs in the elderly should be issued.

## Abbreviations

ADR: adverse drug reaction
HDR: hypersensitivity drug reaction
NSAID: non steroidal anti-inflammatory drugs
ASA: acetylsalicylic acid
BAT: basophil activation test
ACE: angiotensin converting enzyme
